# Regulation of the high-affinity copper transporter (hCtr1) expression by cisplatin and heavy metals

**DOI:** 10.1007/s00775-013-1051-z

**Published:** 2013-10-17

**Authors:** Zheng Dong Liang, Yan Long, Helen H. W. Chen, Niramol Savaraj, Macus Tien Kuo

**Affiliations:** 1 Department of Translational Molecular Pathology (Route 2951), The University of Texas MD Anderson Cancer Center, 2130 W. Holcombe Blvd., Houston, TX 77030 USA; 2 Department of Radiation Oncology, National Cheng Kung University Hospital, College of Medicine, National Cheng Kung University, Tainan, Taiwan; 3 Hematology-Oncology Section, VA Medical Center, Miami, FL 33125 USA

**Keywords:** Cisplatin, High-affinity copper transporter, Sp1, Copper homeostasis, Platinum drug resistance

## Abstract

Platinum-based antitumor agents have been the mainstay in cancer chemotherapy for many human malignancies. Drug resistance is an important obstacle to achieving the maximal therapeutic efficacy of these drugs. Understanding how platinum drugs enter cells is of great importance in improving therapeutic efficacy. It has been demonstrated that human high-affinity copper transporter 1 (hCtr1) is involved in transporting cisplatin into cells to elicit cytotoxic effects, although other mechanisms may exist. In this communication, we demonstrate that cisplatin transcriptionally induces the expression of hCtr1 in time- and concentration-dependent manners. Cisplatin functions as a competitor for hCtr1-mediated copper transport, resulting in reduced cellular copper levels and leading to upregulated expression of Sp1, which is a positive regulator for hCtr1 expression. Thus, regulation of hCtr1 expression by cisplatin is an integral part of the copper homeostasis regulation system. We also demonstrate that Ag(I) and Zn(II), which are known to suppress hCtr1-mediated copper transport, can also induce hCtr1/Sp1 expression. In contrast, Cd(II), another inhibitor of copper transport, downregulates hCtr1 expression by suppressing Sp1 expression. Collectively, our results demonstrate diverse mechanisms of regulating copper metabolism by these heavy metals.

## Introduction

Platinum-based drugs have been the mainstay of cancer chemotherapy for a broad spectrum of human malignancies for the last three decades [1, 2]. However, resistance to these drugs has been an obstacle to their effective use [3–5]. Although many mechanisms have been described for platinum drug resistance, a well-recognized and important mechanism of resistance is the reduced transport or enhanced efflux (or both) of cellular platinum drugs [6, 7].

Multiple mechanisms are involved in platinum drug transport. Cisplatin—*cis*-[PtCl_2_(NH_3_)_2_]—may enter cells by means of passive diffusion or endocytosis [3, 8, 9] and by high-affinity copper transporter 1 (Ctr1). The involvement of Ctr1 in cisplatin transport was initially demonstrated using yeast genetics which showed that deletion of *CTR1* resulted in impaired cisplatin transport and cisplatin resistance [10, 11]. In a *Ctr1*-knockout murine embryonic fibroblast model, although *Ctr1* ^−/−^ cells accumulated only 5.7 % of the amount of copper that *Ctr1* ^+/+^ cells accumulated during 1 h exposure to 2 μM copper, the amount of cisplatin accumulated in these *Ctr1* ^−/−^ cells was 35–36 % of that accumulated in *Ctr1* ^+/+^ cells [8]. Moreover, cisplatin-resistant human cell lines exhibited reduced cisplatin contents, and the resistance was restored when *CTR1* was introduced into these cells [12–14]. These results demonstrated that Ctr1 plays an important role in cisplatin resistance.

In clinical studies, the human Ctr1 (hCtr1) expression level in tumor tissue specimens has been positively correlated with the treatment outcome of patients who had undergone platinum-based cancer chemotherapy [14–16]. Using cultured cell models, we recently demonstrated that copper chelators could upregulate the hCtr1 level to a greater extent in cisplatin-resistant cells than in cisplatin-sensitive cells, leading to resensitization of the resistant cells to cisplatin [14]. These findings provided a mechanistic basis for the first study in humans using a copper chelator to overcome platinum resistance in ovarian cancer patients [17].

The observation that copper chelation enhances hCtr1 expression was part of our previous investigation into the mechanisms of mammalian copper homeostasis regulation. We demonstrated that copper chelation induces the expression of transcription factor Sp1, which binds the promoters of *SP1* and *CTR1*, thereby upregulating their expression, whereas copper overload shuts down expression of *SP1* and *CTR1* by dissociating Sp1 from their promoters. Thus, mammalian copper homeostasis is transcriptionally regulated within a loop consisting of Sp1, hCtr1, and copper in a three-way mutually regulated manner [6, 18]. Posttranslational regulation which involves the internalization and subcellular processing of hCtr1 in response to extracellular copper availability has also been reported [6],

Although much has been learned on the regulation of hCtr1 expression by copper bioavailability, whether hCtr1 is regulated by cisplatin and other metal ions is not known. In this communication, we demonstrate that cisplatin, Ag(I), Zn(II), and Cd (II) can also regulate hCtr1 expression through interference with copper homeostasis, thus revealing a regulatory mechanism of copper homeostasis by cisplatin and heavy metal ions.

## Materials and methods

### Cell lines and reagents

Human ovarian cancer cell lines (IGROV1, SKOV-3, 59M, and OVCAR-3) were obtained from Gordon Mills (MD Anderson Cancer Center). The small cell lung cancer (SCLC) cell line was obtained from N. Savaraj (University of Miami School of Medicine, Miami, FL, USA). Polyclonal anti-hCtr1 antibody obtained using the extracellular 50 amino acid residues of hCtr1 as the immunogen was previously described [14]. Sp1 antibody was obtained from Santa Cruz Biotechnology (Santa Cruz, CA, USA). Cisplatin was purchased from Sigma-Aldrich (St Louis, MO, USA). Other chemicals were of chemical grade.

### Cell culture and determination of hCtr1 and Sp1 messenger RNA and protein expression by the RNase protection assay and Western blotting

Cells were grown in Dulbecco’s modified Eagle’s medium supplemented with 10 % fetal bovine serum at 37 °C in a 5 % CO_2_ atmosphere. Cells at the exponential growth stage were treated with cisplatin, CuSO_4_, AgNO_3_, zinc acetate (ZnAc_2_) or cadmium acetate (CdAc_2_). Procedures for RNA extraction and determination of hCtr1 messenger RNA (mRNA) and Sp1 mRNA levels by the RNase protection assay (RPA) using the isoform-specific probes were described previously [12, 13]. Procedures for Western blotting were described previously [12, 13]. Owing to the constraint of the copper homeostasis regulatory loop [6], the magnitudes of hCtr1 and Sp1 regulation by cisplatin and heavy metals were low at the mRNA and protein levels. Thus, the conditions for the RPA and Western blotting needed to be optimized.

Images were taken only under exponential exposure conditions. Images were scanned in grayscale at a resolution of 600 dpi. The band intensities were measured with ImageJ [19] and normalized using the intensity of tubulin for Western blots or 18S for RPA as references.

All statistical analyses were conducted from at least three measurements using the two-tailed *t* test, and the results were expressed as the mean ± the standard deviation (SD); *p* < 0.05 was considered as statistically significant.

### Copper and cisplatin transport assays, determination of drug sensitivity, and measurement of *K*_m_ and *V*_max_

For copper transport analyses, 2 × 10^5^ cells per well were plated in six-well plates. After 12 h, fresh medium containing various concentrations of CuSO_4_ was added and cultured for various time intervals. Cells were washed four times with phosphate-buffered saline and then lysed in 400 μl of lysis buffer [13]. Cellular copper content was measured using atomic absorption spectroscopy. For cisplatin transport measurement, 5 × 10^6^ cells per well were treated with various concentrations of cisplatin. Cells were harvested and lysed in 50 μl of benzethonium hydroxide at 50 °C for 16 h [13]. The lysates were acidified with 200 μl of 0.3 N HCl, and the platinum content was determined by a Zeeman atomic absorption spectrometer (AA240Z) equipped with a GTA12 graphite atomizer according to the procedure described previously [14]. The results were from at least three measurements and are given as the mean ± the SD.

Drug sensitivity tests were performed according to the procedure described previously [12]. In brief, cells were grown in 96-well plates (10^4^ cells per well) and were treated with various concentrations of cisplatin, CuSO_4_, or CdAc_2_, and cell sensitivity was measured by the 3-(4,5-dimethylthiazol-2-yl)-2,5-diphenyltetrazolium bromide assay. Experiments were performed with eight replicates at each dose. Values represent the mean ± the SD.

Measurements of the *V*_max_ and *K*_m_ values were done according to the procedures previously described [13]. *V*_max_ and *K*_m_ were calculated according to the Michaelis–Menten equation: 1/*V* = 1/*V*_max_ + *K*_m_/(*V*_max_ × [S]), where [S] is the copper or cisplatin concentration, and *V* is the copper or platinum concentration inside the cells at a given time point according to the procedures previously described [13].

## Results

### Upregulation of hCtr1 and Sp1 expression by cisplatin

**Fig. 1:**
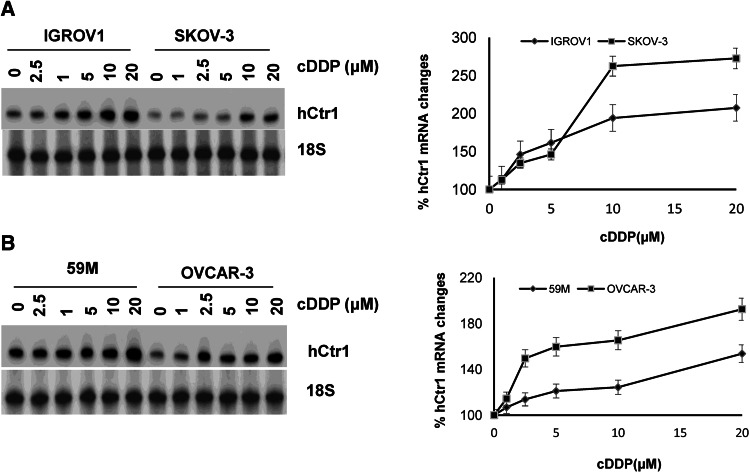
Regulation of human high-affinity copper transporter 1 (*hCtr1*) expression in ovarian cancer cell lines by cisplatin. Induction of hCtr1 messenger RNA (*mRNA*) expression by different concentrations of cisplatin as indicated for 20 h in **a** the IGROV1 and SKOV-3 cell lines and **b** the 59M and OVCAR-3 cell lines. The hCtr1 mRNA levels were determined by the RNase protection assay (RPA), and the percent changes are correspondingly shown on the *right* (*n* = 3). *cDDP* cisplatin

Cisplatin has been the dominant chemotherapeutic drug for treating ovarian cancers [2]. In this work, we used four ovarian cancer cell lines, two from patients who had never been treated with cisplatin (IGROV1 [20] and M59 [21]) and two from cisplatin-relapsed patients (SKOV-3 [22] and OVCAR-3 [23]). We previously demonstrated that SKOV-3 and OVCAR-3 express reduced hCtr1 mRNA levels as compared with those in IGROV1 and 59M [14]. To investigate whether hCtr1 expression is regulated by cisplatin in these cell lines, we grouped these four cell lines into two pairs, i.e., IGROV1 vs SKOV-3 and 59M vs OVCAR-3; each pair consisted of one high hCtr1 expresser (IGROV1 and 59M) and one low hCtr1 expresser (SKOV-3 and OVCAR-3). We treated these cells with various concentrations of cisplatin for 20 h, and RPA was used to determine hCtr1 mRNA levels in the treated cells. We found that in all four cell lines, a cisplatin-concentration-dependent increase of hCtr1 mRNA expression was observed, and that the magnitudes of hCtr1 mRNA induction by cisplatin were higher in cells expressing reduced levels of hCtr1 mRNA than in cells expressing elevated levels of hCtr1 mRNA. Densitometric analyses showed that OVCAR-3 cells, which express the lowest level of hCtr1 of the cell lines tested, had the highest level (2.5-fold) of hCtr1 mRNA induction, whereas 59M cells, which have the highest level of hCtr1, had the lowest level (1.4-fold) (Fig. 1, a, b, right). These results demonstrated that cells with reduced hCtr1 expression levels have greater magnitudes of hCtr1 induction by cisplatin than do those expressing reduced levels of hCtr1. These results are consistent with those of our previous study using copper-lowering agents [14].

**Fig. 2:**
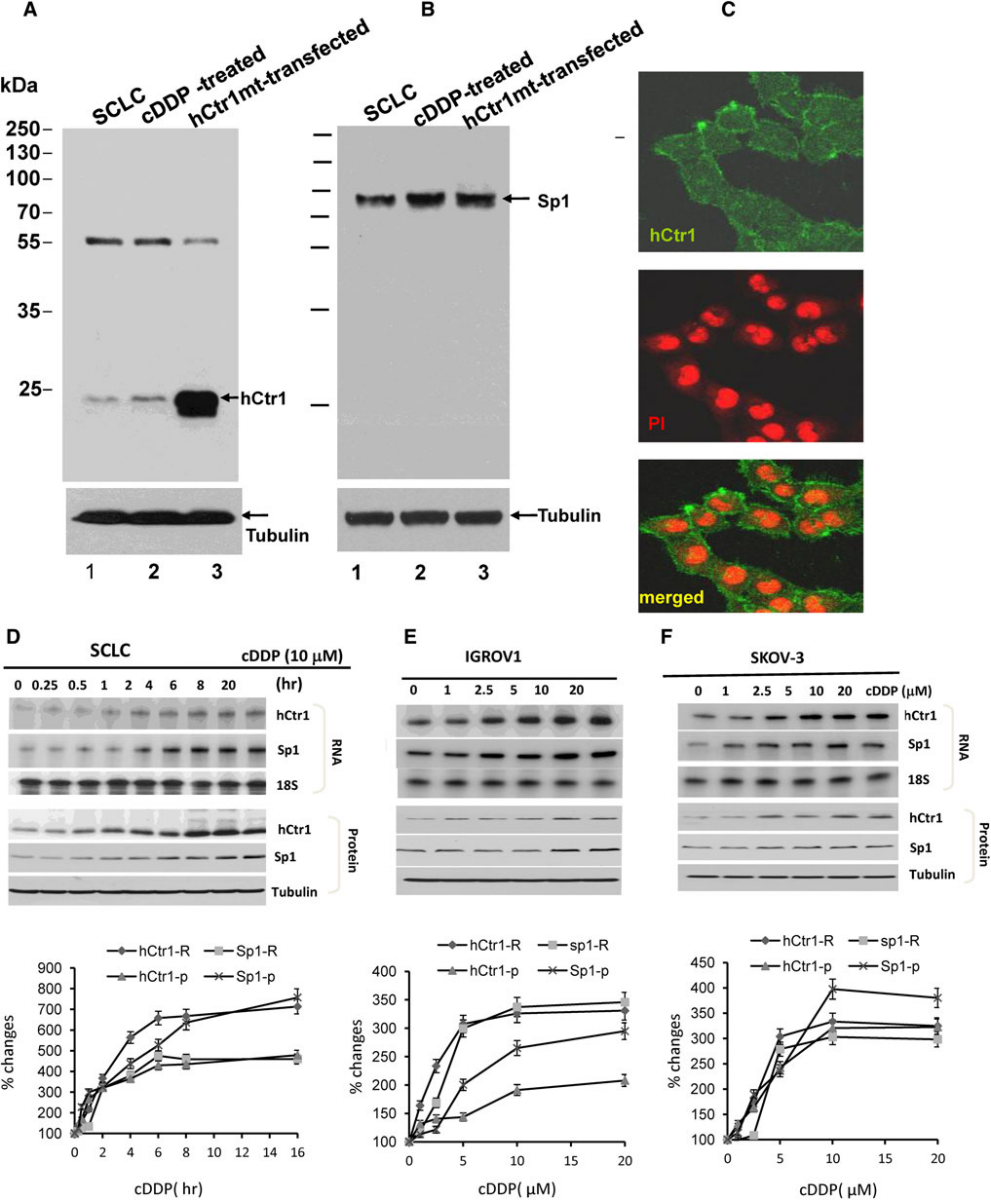
Characterizations of anti-hCtr1 antibody and analysis of hCtr1 induction by cisplatin. **a**, **b** Western blot of cell extracts prepared from different cell sources as indicated using anti-hCtr1 antibody (**a**) and anti-Sp1 antibody (**b**). **c** Immunofluorescence images of hCtr1 detected by anti-hCtr1 antibody (*top*) and the same cells stained by propidium iodide (*PI*) (*middle*), and the merged image (*bottom*). **d** Time-dependent induction of hCtr1 and Sp1 expression by cisplatin. Small cell lung cancer (*SCLC*) cells were treated with 10 μM cisplatin for the times indicated. Expression levels of hCtr1 and Sp1 mRNA and protein were determined by the RPA (*top three rows*) and Western blotting (*bottom three rows*), respectively. **e**, **f** Concentration-dependent induction of IGROV1 or SKOV-3 cells treated with various concentrations of cisplatin as indicated for 16 h. Expression of hCtr1 and Sp1 mRNA and protein was similarly determined. Densitometric analyses of the expression levels shown in **d**–**f** are correspondingly shown below the Western blots (*n* = 3). *cDDP* cisplatin

We next investigated whether upregulation of hCtr1 mRNA by cisplatin could be seen at the protein level using anti-hCtr1 antibody. This antibody was prepared in our laboratory in 2010 and has been kept at −80 °C [14]. To determine whether this antibody was still reliable for probing hCtr1 protein expression, we performed Western blotting using whole cell extracts prepared from SCLC cells and cisplatin-treated SCLC cells, and SCLC cells transfected with a dominant-negative mutant *CTR1* complementary DNA (a positive control for hCtr1 expression). Consistent with our previous findings [14], this antibody reacts with two proteins (approximately 55 and 23 kDa) (Fig. 2a), but only the 23-kDa signal, which corresponds to the molecular mass of an unmodified hCtr1 monomer, was increased in the cisplatin-treated cells and the *CTR1*-transfected cells. This antibody stains the cell membrane with minor cytoplasmic staining, consistent with the primary cytologic location of hCtr1 (Fig. 2c). These results demonstrated that the hCtr1 antibody was still reliabe for Western blotting.

We also determined the expression levels of Sp1 in these cell extracts by using a commercial antibody. Anti-Sp1 antibody detected only a 98-kDa signal, consistent with the molecular mass of Sp1. The signals were also increased in cisplatin-treated SCLC cells (Fig. 2b), and in *CTR1*-transfected SCLC cells owing to expression of the dominant-negative hCtr1 recombinant, which acts much like a copper-lowering agent [14, 18].

Protein and mRNA levels of hCtr1 and Sp1 in SCLC cells treated with 10 μM cisplatin for various times were determined by the RPA (Fig. 2d, top) and Western blotting (Fig. 2d, bottom). Upregulation of hCtr1 and Sp1 mRNA occurred between 0.5 and 2 h after the treatment and plateaued 6–8 h later with approximately fourfold to sevenfold increases. Sp1 and hCtr1 mRNA and protein induced by cisplatin followed the same kinetics, suggesting that the induction is coordinated. These results demonstrated that the regulation of hCtr1 and Sp1 expression by cisplatin is mainly at the mRNA level.

We observed a concentration-dependent induction of hCtr1 and Sp1 expression in IGROV1 cells (Fig. 2e) and SKOV-3 cells (Fig. 2f) treated with cisplatin for 16 h. Induction of hCtr1 and Sp1 expression could be seen at 2.5 μM, a concentration relevant to the therapeutic dose. Induction of hCtr1 and Sp1 expression was generally correlated at the mRNA and protein expression levels. Thus, from the investigation of five cell lines, we concluded that hCtr1 and Sp1 are coordinately upregulated by cisplatin in human cancer cells.

### Induction of hCtr1 by cisplatin is regulated by Sp1

**Fig. 3:**
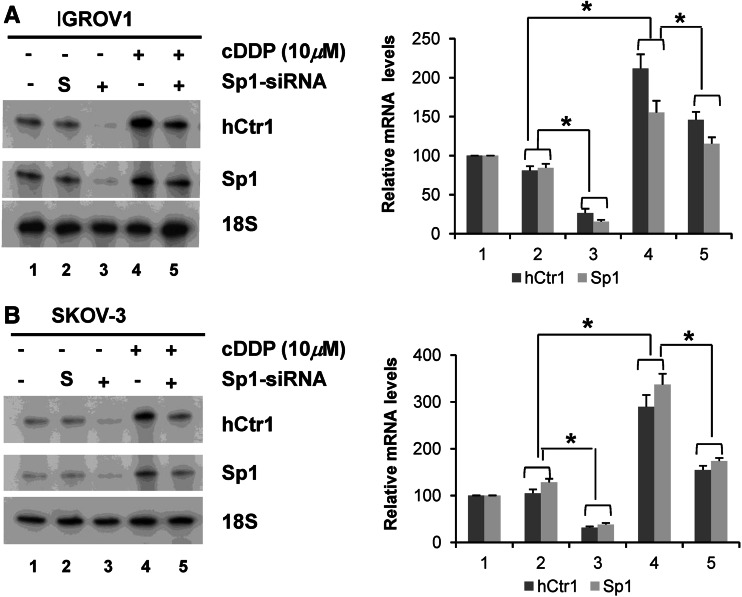
Knockdown of Sp1 suppresses cisplatin-induced hCtr1 expression. IGROV1 (**a**) and SKOV-3 (**b**) cells were treated with or without 10 μM cisplatin for 16 h in the presence of scrambled small interfering RNA (*siRNA*) (denoted by *S*) or Sp1 siRNA as indicated. Levels of hCtr1 mRNA were determined by the RPA using 18S ribosomal RNA as a reference (*left*). Densitometric measurements of percent changes are shown on the *right*. *Asterisk* *p* < 0.05 by Student’s *t* test (*n* = 3), *cDDP* cisplatin

To investigate whether enhanced hCtr1 expression is regulated by co-induced Sp1, we treated IGROV1 and SKOV-3 cells with Sp1 small interfering RNA (siRNA) followed by 10 μM cisplatin for 16 h. Knockdown of Sp1 by siRNA almost completely suppressed the expression of hCtr1 mRNA, but hCtr1 mRNA levels remained unchanged in cells treated with scrambled siRNA (Fig. 3a, b, left, lane 2). Treating cells with cisplatin induced hCtr1 and Sp1 expression, and Sp1 siRNA suppressed the induction of both in both cell lines. These results demonstrated that the upregulation of hCtr1 by cisplatin is controlled by Sp1.

### Suppression of cisplatin-induced hCtr1 and Sp1 expression by copper

We hypothesized that induction of hCtr1 and Sp1 by cisplatin was due to competition for hCtr1-mediated copper transport by cisplatin resulting in reduced cellular copper levels. Under this scenario, cisplatin functions like a copper-lowering agent for Sp1/hCtr1 induction. Although reduced copper levels can be verified by reduced activities of copper-dependent enzymes, such as superoxide dismutase 1 (SOD1) and mitochondrial cytochrome *c* oxidase (CCO) [24, 25], we considered that these biomarkers are inadequate for determining cellular copper status in the cisplatin-treated cells, because SOD1 and CCO are redox-sensitive enzymes [26] and cisplatin is a potent redox-generating agent [27]. Consistent with this notion, we found that the antitumor drug elesclomol (STA4783), which is a strong pro-oxidant copper chelator [28], induced hCtr1 expression but that SOD1 and CCO activities were also increased (our unpublished data). Ceruloplasmin, another frequently used copper biomarker [24, 25], would not be suitable because cisplatin can also bind ceruloplasmin [29].

**Fig. 4:**
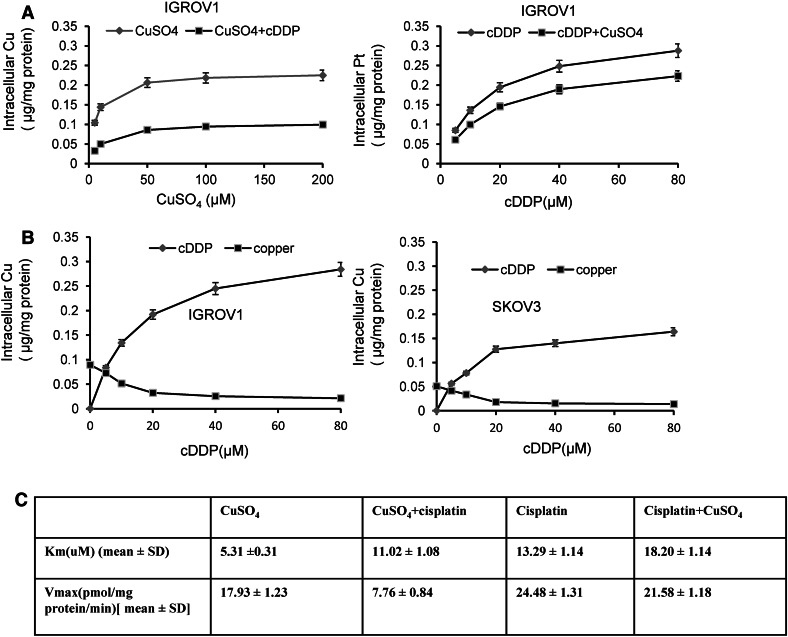
Reciprocal inhibition of copper and cisplatin uptake. **a** *Left* inhibition of copper uptake by cisplatin in IGROV1 cells. Cells were treated with different concentrations of CuSO_4_ as indicated alone or with 10 μM cisplatin for 2 h. Intracellular copper contents were determined by atomic absorption spectroscopy. **a** *Right* inhibition of cisplatin uptake by CuSO_4_. IGROV1 cells were treated with different concentrations of cisplatin as indicated alone or with 100 μM CuSO_4_ for 2 h. Intracellular platinum contents were determined by atomic absorption spectroscopy. **b** Inhibition of cellular copper accumulation by cisplatin in IGROV1 cells (*left*) and SKOV-3 cells (*right*) cells. Cells were treated with different concentrations of cisplatin for 4 h. Cellular copper and cisplatin were measured. **c** Kinetic parameters for copper and cisplatin uptake alone or in combination in IGROV1 cells. The values represent the mean ± the standard deviation (*SD*) (*n* = 3). *cDDP* cisplatin

To demonstrate that cisplatin competes against copper in hCtr1-mediated transport, we determined the uptake of copper in the presence of cisplatin and vice versa. Treating IGROV1 cells with increased concentrations of copper resulted in increased cellular copper levels, but these were suppressed by the addition of 10 μM cisplatin (Fig. 4a, left). Likewise, treating IGROV1 cells with increased concentrations of cisplatin increased its uptake, and addition of 100 μM CuSO_4_ suppressed its transport (Fig. 4a, right).

To determine whether cisplatin treatment would reduce the steady-state levels of cellular copper levels, we treated IGROV1 and SKOV-3 cells with various concentrations (5–80 μM) of cisplatin for 4 h. Figure 4b shows that reduced cellular copper contents correspond to increased concentrations of cisplatin in both cell lines. These results suggest that cisplatin treatment resulted in reduced copper accumulation.

To more precisely determine how cisplatin affects hCtr1-mediated Cu(I) transport and vice versa, we measured the kinetic constants *K*_m_ and *V*_max_ in IGROV1 cells. *K*_m_ for copper uptake was 5.31 ± 0.31 μM (Fig. 4c), which is in agreement with the values reported for Ctr1-mediated Cu(I) transporters in a variety of organisms (between 1 and 6 μM) [13, 30, 31]. In the presence of cisplatin, however, *K*_m_ increased to 11.02 ± 1.08 μM (about a twofold increase), whereas *V*_max_ reduced from 17.93 ± 1.33 to 7.76 ± 0.84 pmol/mg protein/min (more than twofold reduction), indicating that cisplatin interferes with hCtr1-mediated Cu(I) transport. *K*_m_ for cisplatin uptake was 13.29 ± 1.14 μM, which is also in agreement with the previous result (11–13 μM) [13]. In the presence of CuSO_4_, *K*_m_ for cisplatin uptake was 18.20 ± 1.14 μM (a 37 % increase), whereas *V*_max_ decreased from 24.48 ± 1.31 to 21.58 ± 11.18 pmol/mg protein/min, only 11 % reduction. These results further suggest that cisplatin and Cu(I) mutually interfere with each other in their transport [32] and strongly support the notion that cisplatin is a weak substrate of hCtr1.

**Fig. 5:**
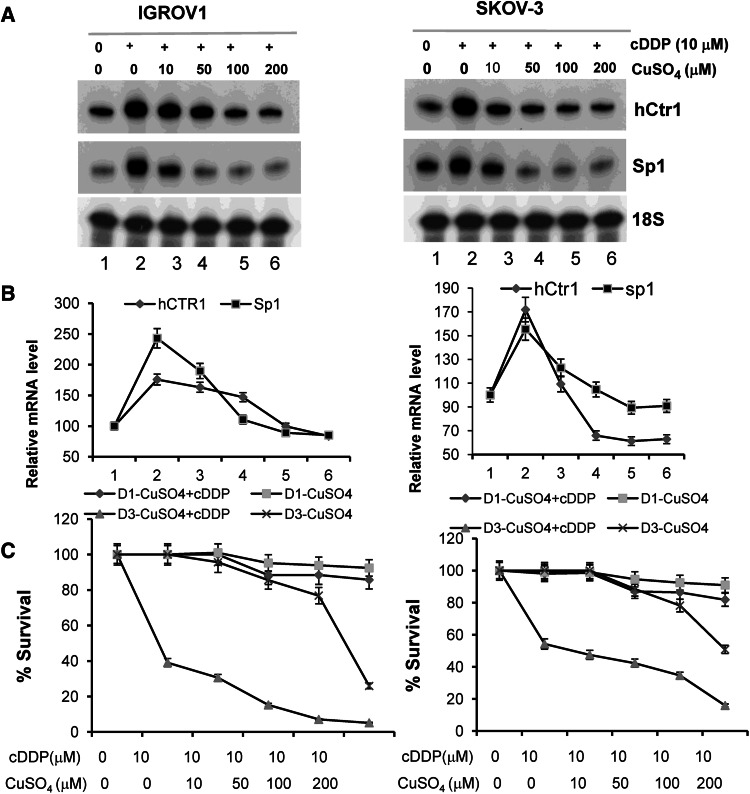
Effect of copper on cisplatin-induced hCtr1 and Sp1 mRNA expression and cytotoxicity in cultured cells. **a** IGROV1 and SKOV-3 cells were treated with 10 μM cisplatin for 16 h in the presence of various concentration of CuSO_4_. Sp1 and hCtr1 mRNA levels were determined by the RPA. **b** Densitometric measurements of the RPA results shown in **a** (*n* = 3). **c** Cytotoxicity tests of IGROV1 and SKOV-3 cells treated with cisplatin alone or cisplatin plus increased concentrations of CuSO_4_, or different concentrations of CuSO_4_ without cisplatin for 16 h (D1 series) or for 3 days (D3 series). Cytotoxicities were determined by the 3-(4,5-dimethylthiazol-2-yl)-2,5-diphenyltetrazolium bromide assay (mean ± SD, eight replicates in each dose). *cDDP* cisplatin

To substantiate these results, we investigated whether upregulation of hCtr1 and Sp1 could be suppressed by CuSO_4_. IGROV1 and SKOV-3 cells were treated with 10 μM cisplatin in the presence of increased concentrations of CuSO_4_ for 16 h. We found approximately 2.5-fold and 1.7-fold induction of hCtr1 and Sp1 mRNA, respectively, in cells treated with cisplatin alone. SP1 and hCtr1 mRNA expression levels were reduced 50–70 % when 50 μM CuSO_4_ was included, and were completely suppressed when 100 or 200 μM CuSO_4_ was used (Fig. 5a, b, lanes 5 and 6). These results demonstrated that induction of hCtr1/Sp1 expression by cisplatin is due to reduced cellular copper content.

We next investigated whether inhibition of cisplatin-induced hCtr1 expression by copper would reduce cisplatin’s cell-killing capacity. We measured the survival rates of IGROV1 and SKOV-3 cells treated with 10 μM cisplatin plus increased concentrations of CuSO_4_ (like the treatment shown in Fig. 5a), the same concentration range of CuSO_4_ without cisplatin, or 10 μM cisplatin alone for 16 h. Treating IGROV1 and SKOV-3 cells with 10 μM cisplatin alone resulted in survival rates of 92.9 ± 4.7 and 95.3 ± 4.8 %, respectively. The survival rates decreased in a concentration-dependent manner in IGROV1 and SKOV-3 cells treated with 10 μM cisplatin plus 200 μM CuSO_4_, reaching 72.9 ± 4.1 and 75.9 ± 5.2 %, respectively, whereas they were 81.8 ± 4.1 and 85.7 ± 5.2 %, respectively, for treatment with 200 μM CuSO_4_ alone (Fig. 5c).

Treating IGROV1 and SKOV-3 cells with 200 μM CuSO_4_ for 3 days resulted in survival rates of 25.9 ± 0.6 and 50.8 ± 0.16 %, respectively. In the presence of 10 μM cisplatin, the survival rates correspondingly reduced to 5.1 ± 0.12 and 15.9 ± 0.12 %, respectively (Fig. 5c). These results demonstrated that despite the suppression of cisplatin-induced hCtr1 mRNA expression by copper as shown in Fig. 5a, no corresponding reduction of cellular toxicity was found.

### Regulation of hCtr1 and Sp1 expression by metal ions

**Fig. 6:**
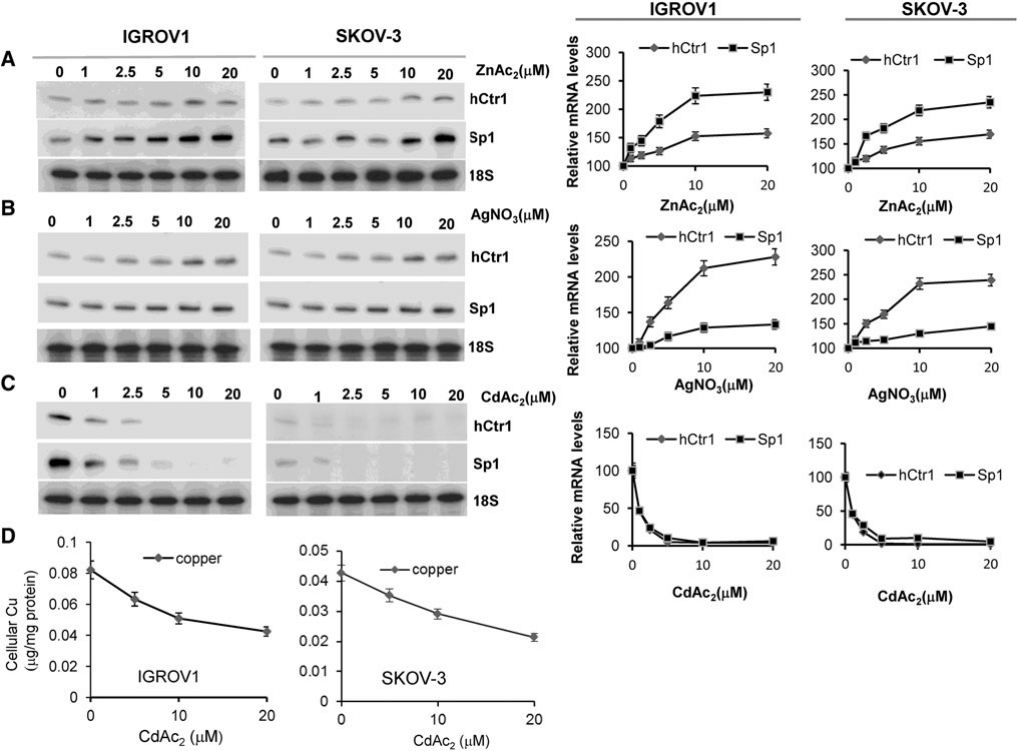
Induction of hCtr1 and Sp1 mRNA expression by different metal ions. IGROV1 and SKOV-3 cells were treated with zinc acetate (*ZnAc*_*2*_) (**a**), AgNO_3_ (**b**), and cadmium acetate (*CdAc*_*2*_) (**c**) at the concentrations indicated for 16 h. Expression of hCtr1 and Sp1 mRNA was determined by the RPA. Corresponding relative levels of induction (as a percentage) are shown on the *right*. **d** Suppression of copper uptake by CdAc_2_. IGROV1 cells (*left*) or SKOV-3 cells (*right*) were treated with different concentrations of CdAc_2_ for 16 h. Cells were harvested and cellular copper levels were determined (*n* = 3)

A previous study demonstrated that Ag(I), Zn(II), and Cd(II) ions inhibited hCtr1-mediated Cu(I) uptake in hCtr1-transfected or nontransfected HEK293 cells [33]. However, the underlying mechanisms were not investigated. We determined the expression of hCtr1 and Sp1 mRNA in IGROV1 and SKOV-3 cells treated with different concentrations of ZnAc_2_, AgNO_3_, or CdAc_2_ for 16 h. We found concentration-dependent increased expression of hCtr1 and Sp1 by ZnAc_2_ (Fig. 6a) and AgNO_3_ (Fig. 6b), with maximal induction levels of approximately twofold for hCtr1 mRNA but 1.2-fold to 1.5-fold for Sp1 mRNA. In contrast, even 1 μM CdAc_2_ suppressed the expression of hCtr1 and Sp1 mRNA (Fig. 6c). Suppression of hCtr1 expression by CdAc_2_ corresponded to reduction of copper levels (Fig. 6d). These results suggest that Ag(I) and Zn(II), like cisplatin, function as competitors for hCtr1-mediated Cu(I) transport. In contrast, inhibition of copper uptake by Cd(II) was associated with downregulation of hCtr1 resulting from inhibition of Sp1 expression. We and others previously demonstrated that Cd(II) transport is mainly mediated by ZIP8 (SLC39A8) [34, 35]. Here, we demonstrated that inhibition of copper uptake by Cd(II) is due to suppression of hCtr1 expression through the downregulation of Sp1. These results revealed diverse mechanisms of inhibition of hCtr1-mediated copper transport by these heavy metal ions.

## Discussion

The major finding of this study is that cisplatin induces hCtr1 expression through the co-induced expression of Sp1, which functions as a positive regulator for hCtr1 expression. This finding reveals a new role of hCtr1 in the cisplatin pharmacology and provides important insights into how cisplatin may use hCtr1 in eliciting its antitumor activity in cancer chemotherapy.

Induction of hCtr1 by cisplatin treatment could be seen at the mRNA and protein levels. Although the magnitudes of induction were small, the results were highly reproducible. Changes of hCtr1 expression were demonstrated in concentration- and time-dependent manners. The small magnitudes of the changes reflect the tightly controlled copper homoeostasis regulation mechanism [6]. Our ability to detect small changes relied on the use of appropriate assay tools: an isoform-specific RPA probe for mRNA and a well-characterized anti-hCtr1 antibody in Western blotting. Polyclonal anti-hCtr1 antibodies reported by different laboratories detected different molecular masses of hCtr1 monomer, i.e., 28 kDa [36], 24 kDa [37], 35 kDa [38], and 24 and 36 kDa [39]. The 23–24-kDa protein corresponds to the unmodified hCtr1 monomer, which consists of 190 amino acid residues. The 35–36-kDa protein is considered to be glycosylated hCtr1 [24, 38]. Although the roles of glycosylated hCtr1 and native hCtr1 in copper transport remain to be critically investigated, we demonstrated in this and previous communications [14] that the expression levels of 23-kDa hCtr1 are well correlated with hCtr1 mRNA levels within the context of overall copper homeostasis regulation. However, the antibody that recognizes 35-kDa hCtr1 failed to detect hCtr1 changes in human embryonic kidney cells that overexpressed hCtr1 by transfection [40]. Because our antibody recognizes unmodified hCtr1 monomer, the signal shown in the Western blot is discrete, whereas the 35-kDa signal is general is more diffuse because of different degrees of protein modification. The discrete signal can be readily quantified as demonstrated in this study.

The demonstration that cisplatin regulates hCtr1 expression as an integral part of mammalian copper homeostasis regulation lends further support for the importance of hCtr1 in cisplatin transport. The controversy surrounding cisplatin as a substrate for hCtr1 relates to its molecular size and charge [40]. Although the molecular size of cisplatin is greater than the “narrowest opening” of trimeric hCtr1, recent studies suggest that prior to entering a cell, cisplatin is activated by interacting with the extracellular methionine clusters of hCtr1, resulting in release of the carrier ligand [41–43] and formation of the [Pt(Met)Cl(NH_3_)_2_]^+^ intermediate. The radius of this platinum intermediate (2.4 Å) [44] is smaller than the radius of the narrowest opening (4 Å) of hCtr1 [45]. Moreover, we previously demonstrated that cisplatin, like copper, can induce conformational stabilization of trimeric hCtr1 [13]. The dynamic nature of the interactions between cisplatin and its transporter has to be taken into consideration when modeling the molecular basis of hCtr1-mediated transport. Extracellular copper exists in an oxidized form (Cu^2+^) but is reduced to Cu^+^ by the membrane-bound cupric reductases [46] before transport. Thus, the activated cisplatin intermediate also shares a similar charge content as in Cu^+^. It has been proposed that the activated cisplatin may use methionine-based intermolecular sulfur–sulfur exchange along the axis of trimeric hCtr1 to facilitate its transport [44], much like the mechanism underlying Cu(I) or Ag(I) transport by the CusA efflux pump in bacteria [47]. This mechanism is consistent with the recently proposed model describing how Cu(I) ions pass through the transporter [7, 48]. Given that hCtr1-mediated cisplatin transport shares many similarities with transport of copper, including most of the proposed binding sites [13], it is likely that the proposed model could be shared by cisplatin transport.

Upregulated hCtr1 and accumulation of cellular cisplatin were seen in cisplatin-treated cells (see Fig. 4a, left). However, it is difficult to assign the extent by which upregulated hCtr1 contributed to the overall cellular platinum accumulation, because the level of cisplatin (which is a substrate) was also increased in the system. Induction of hCtr1 expression by a copper-lowering agent takes several hours to reach a plateau, but requires 72–96 h to return to its basal level after the agent is removed [14]. We also showed that treating cells with CuSO_4_ resulted in accumulation of cellular copper content (see Fig. 4a, right), whereas expression of hCtr1 was downregulated in cells treated with copper [18, 49]. A plausible explanation to account for these results is increased hCtr1 transport activity, because expression of copper efflux transporters (ATP7A and ATP7B) is not regulated by acute environmental copper changes [6, 14]. These results demonstrated that the capacities of cellular copper or cisplatin accumulations cannot be simply related to the abundance of hCtr1 when cells are exposed to its substrates; rather, they are controlled by the overall copper homeostasis system, which involves interregulatory components consisting of copper, hCtr1, and Sp1 [6]. Consistent with this model, we showed that although cisplatin treatment induced hCtr1 expression, cellular copper levels were reduced (Fig. 4b), suggesting that cisplatin is a competitor for hCtr1-mediated copper transport.

This argument may explain why addition of copper suppressed cisplatin-induced hCtr1 expression yet no reduction in overall cytotoxicity was found. Furthermore, both cisplatin and copper are potent pro-oxidants with many cellular lethality targets. The complexity of the cytotoxic mechanisms of cisplatin and copper, especially when in combination, cannot be explained by the expression of hCtr1 alone.

We found that Ag(I) and Zn(II) can also induce Sp1/hCtr1 expression, which can be explained by the same mechanism in that these metals may compete against hCtr1-mediated copper transport, resulting in reduced cellular copper levels and thereby upregulated Sp1/hCtr1. Because the major influx transporter for Zn(II) is the SLC39 family [50], the physiological implication of hCtr1 upregulation by Zn(II) remains to be evaluated. However, it has been reported that zinc supplement can enhance the treatment efficacy of platinum-based chemotherapy in nasopharyngeal carcinoma [51]. Likewise, several organs in silver-fed mice exhibited rates of cisplatin uptake different from those in normal-diet-fed mice [52], suggesting that Ag(I) may affect the pharmacodynamics of platinum drugs in cancer chemotherapy. In contrast, we found that Cd(II) suppresses Sp1 expression (and therefore hCtr1 expression), most likely owing to Cd(II)-induced damage to the DNA-binding zinc finger motifs of Sp1 [34]. Cd(II) is a potent environmental poison, as are Ag(I) and Zn(II) to lesser extents. The current study provides important insights into how these heavy metals may affect copper metabolism through interventions in copper homeostasis regulation. 

## Abbreviations

CCO: Cytochrome *c* oxidase
CdAc_2_: Cadmium acetate
Ctr1: High-affinity copper transporter 1
hCtr1: Human high-affinity copper transporter 1
mRNA: Messenger RNA
RPA: RNase protection assay
SCLC: Small cell lung cancer
SD: Standard deviation
siRNA: Small interfering RNA
SOD1: Superoxide dismutase 1
ZnAc_2_: Zinc acetate
